# Active Invasion of Oral and Aortic Tissues by *Porphyromonas gingivalis* in Mice Causally Links Periodontitis and Atherosclerosis

**DOI:** 10.1371/journal.pone.0097811

**Published:** 2014-05-16

**Authors:** Irina M. Velsko, Sasanka S. Chukkapalli, Mercedes F. Rivera, Ju-Youn Lee, Hao Chen, Donghang Zheng, Indraneel Bhattacharyya, Pandu R. Gangula, Alexandra R. Lucas, Lakshmyya Kesavalu

**Affiliations:** 1 Department of Periodontology, University of Florida, Gainesville, Florida, United States of America; 2 Department of Oral Biology, University of Florida, Gainesville, Florida, United States of America; 3 Department of Periodontology, School of Dentistry Pusan National University, Yangsan City, Republic of Korea; 4 Department of Cardiovascular Medicine and Molecular Genetics & Microbiology, University of Florida, Gainesville, Florida, United States of America; 5 Department of Oral Diagnostic Sciences, University of Florida, Gainesville, Florida, United States of America; 6 Department of Physiology, Oral Biology and Research, CWHR Meharry Medical College, Nashville, Tennessee, United States of America; University of Toronto, Canada

## Abstract

Atherosclerotic vascular disease is a leading cause of myocardial infarction and cerebrovascular accident, and independent associations with periodontal disease (PD) are reported. PD is caused by polymicrobial infections and aggressive immune responses. Genomic DNA of *Porphyromonas gingivalis*, the best-studied bacterial pathogen associated with severe PD, is detected within atherosclerotic plaque. We examined causal relationships between chronic *P. gingivalis* oral infection, PD, and atherosclerosis in hyperlipidemic ApoE^null^ mice. ApoE^null^ mice (n = 24) were orally infected with *P. gingivalis* for 12 and 24 weeks. PD was assessed by standard clinical measurements while the aorta was examined for atherosclerotic lesions and inflammatory markers by array. Systemic inflammatory markers serum amyloid A, nitric oxide, and oxidized low-density lipoprotein were analyzed. *P. gingivalis* infection elicited specific antibodies and alveolar bone loss. Fluorescent *in situ* hybridization detected viable *P. gingivalis* within oral epithelium and aorta, and genomic DNA was detected within systemic organs. Aortic plaque area was significantly increased in *P. gingivalis*-infected mice at 24 weeks (*P*<0.01). Aortic RNA and protein arrays indicated a strong Th2 response. Chronic oral infection with *P. gingivalis* results in a specific immune response, significant increases in oral bone resorption, aortic inflammation, viable bacteria in oral epithelium and aorta, and plaque development.

## Introduction

Atherosclerotic vascular disease (ASVD) is the leading cause of death in the U.S, and one third of Americans have some form of the disease, which includes coronary disease with myocardial ischemia, cerebrovascular disease with strokes, and peripheral arterial disease with gangrene [1]. However, half of those with the disease do not have traditional disease risk factors such as obesity, hypercholesterolemia, hypertension, history of smoking, or genetic background [2]–[4], and thus the cause(s) of rapid atherosclerotic plaque progression and disease is unknown in many patients. Prior observational studies have detected or reported a positive correlation between periodontal disease (PD) and ASVD [1], [3], [5], thus PD is proposed as an unrecognized risk factor for ASVD [1]. A recent statement by the American Heart Association supports an association between PD and ASVD that is independent of known confounders, but this report has also stated that current data are insufficient to support a causal relationship [1]. With these studies we examine a potential correlation between chronic infection with a known predominant oral pathogen seen in PD and accelerated atherosclerotic plaque growth using a mouse model for PD and ASVD.

In light of observational studies supporting an association between PD and ASVD, several studies have attempted to demonstrate the presence of periodontal bacteria or their components in human atherosclerotic lesions. *Porphyromonas gingivalis* is a Gram-negative periodontal pathogen, which is implicated in ASVD [1], [6]–[8]. *P. gingivalis* genomic DNA has been detected in some human cardiovascular disease tissues by PCR [9], [10], although not all attempts to isolate bacterial DNA from human clinical atheroma samples have been successful [11], [12]. Live *P. gingivalis* bacteria have also been detected in human atherosclerotic plaque by fluorescence *in situ* hybridization (FISH) [13], invasion assays [14], and culture [15], indicating metabolically active organisms, which are able to invade, survive, and replicate within atheromatous plaques.

Whether *P. gingivalis* plays a direct role in atherosclerotic plaque development is, however, not yet proven as the incidental finding of bacterial organisms or genomes in atheroma may not necessarily demonstrate a true causal relationship. Numerous virulence factors, including bacterial proteases, capsule, and fimbriae allow *P. gingivalis* to modulate its environment to support bacterial growth and invasion [16], and enable *P. gingivalis* to invade gingival epithelial cells *in vivo* and *in vitro* [17], [18], as well as human coronary artery endothelial cells *in vitro* [19]. Finally, *in vivo* studies with ApoE^null^ mice have demonstrated that acute infection with *P. gingivalis* results in larger atherosclerotic plaques [6], [8], [20], but these prior studies did not provide evidence of direct bacterial involvement. Thus, *P. gingivalis* may have a direct effect on atherosclerotic plaque formation by invasion of antigen presenting cells in blood or epithelial cells lining the blood vessel walls, or an indirect effect by increasing soluble inflammatory mediators [21], [22]. The presence of metabolically active bacteria within atherosclerotic lesions points to a direct effect, which may begin with infection-induced endothelial dysfunction as a result of altered nitric oxide (NO) production, which also has a central role in vasodilation, inflammation, thrombosis, and immunity [23]–[25].

The aim of our study was to use a chronic oral infection with the pathogen *P. gingivalis* to investigate the role of viable *P. gingivalis* in the development and progression of atherosclerotic plaque. This study used an ApoE^null^ mouse model with chronic oral administration of *P. gingivalis* to demonstrate that long-term PD allows constant exposure of periodontal bacteria to systemic circulation. *P. gingivalis* interactions with blood vessel endothelial cells and the arterial wall, NO and inflammatory responses, and finally atherosclerotic plaque formation were examined and correlated with aggressive periodontal disease.

## Materials and Methods

### Bacterial strain and growth conditions

*Porphyromonas gingivalis* FDC 381 was grown at 37°C for 2 days, as previously described [26]. Bacteria were suspended in reduced transport fluid (RTF) −4% carboxymethylcellulose (CMC) for oral infection [27].

### Mouse strain and infection

**Figure 1:**
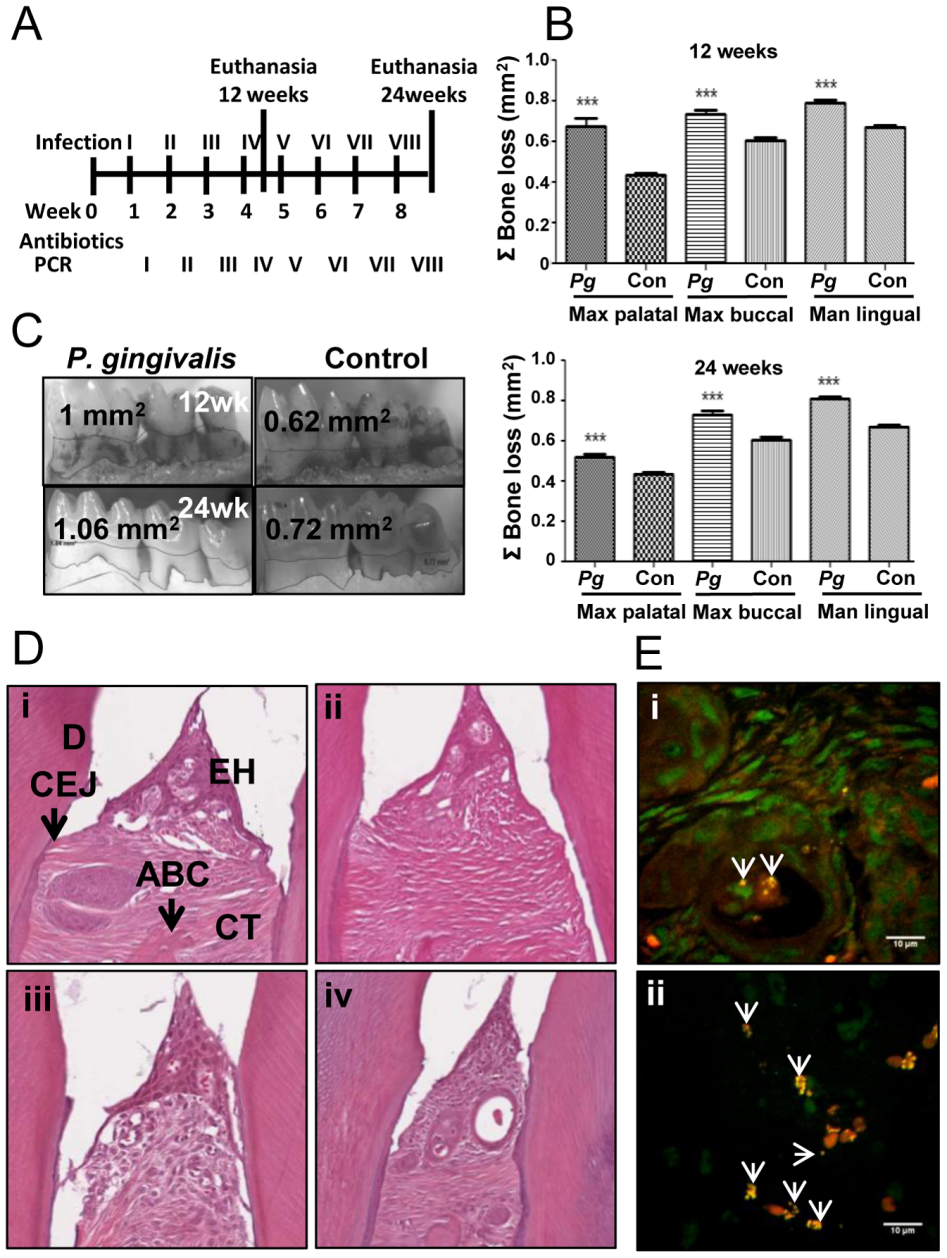
Chronic oral *P. gingivalis* infection and active tissue invasion induced significant alveolar bone resorption. (A) Infection and oral microbial sampling schedule. (B) *P. gingivalis* infected mice (n = 6) had significantly higher levels of alveolar bone resorption relative to control mice (n = 6) at both 12- and 24 weeks of infection (***P<0.001). (C) Mandible lingual view of 12 and 24 week infected and control mice showing outlined area of bone resorption between cemento-enamel junction and alveolar bone crest. The area of bone resorption is across molar 1. (D) Gingival tissue histology. Mild epithelial hyperplasia and minimal inflammation are seen in both 12- (i)- and 24 (iii)-week *P. gingivalis*-infected mice relative to sham-infected age-matched controls (ii and iv, respectively). EH – epithelial hyperplasia, CEJ – cemento-enamel junction, ABC – alveolar bone crest, D – dentin, CT – connective tissue. (E) FISH demonstrates viable *P. gingivalis* invasion of gingival connective tissue in a 12 week-infected mouse (i) and of gingival epithelium in a 24 week-infected mouse (ii). White arrowheads indicate fluorescently labeled bacteria. Scale bar represents 10 µm.

Eight-week old male ApoE^null^ B6.129P2-*Apoe^tm1Unc^*/J mice were obtained from the Jackson Laboratories (Bar Harbor, ME). Mice were acclimated as described [26]. Twenty-four mice were randomly assigned to the infection group, and 24 to the control group. Mice were orally inoculated by lavage with 10^9^ *P. gingivalis* cells in RTF-4% CMC four consecutive days per week every third week for four (12 week) or eight (24 week) weeks of infection (Figure 1A). Sham-infected control mice were mock infected with RTF-4% CMC only. Blood and tissues were collected following euthanasia at 12 and 24 weeks. All experimental procedures were conducted in accordance with the guidelines of the University of Florida Institutional Animal Care and Use Committee (IACUC, protocol #201304539).

### Detection of genomic DNA

Oral microbial samples were taken one week after each infection with a sterile cotton swab, and stored in 150 µl TE (Tris-EDTA) buffer. Samples were boiled for 5 min. The resulting lysate was used directly as template in a PCR reaction as previously described [27]. Tissues for DNA analysis were collected at sacrifice and stored in RNA*later* at −80°C. Later they were homogenized with a QIAGEN TissueRuptor and genomic DNA extracted with the QIAGEN DNEasy Blood and Tissue kit (QIAGEN, Valencia, CA). All PCR was performed as previously described [27] using the following primers, which detect *P. gingivalis*-specific 16S rDNA: forward 5′- GGT AAG TCA GCG GTG AAA CC-3′, reverse 5′- ACG TCA TCC ACA CCT TCC TC-3′ (Integrated DNA Technologies) [27].

### Alveolar Bone Histomorphometric Analysis

Histomorphometric analysis of alveolar bone resorption was performed as follows. The mandibles and maxillas were collected on sacrifice, autoclaved, mechanically defleshed, and immersed in 3% (v/v) hydrogen peroxide for 12 h. Tooth cementum was stained with 0.1% methylene blue and jaws were photographed using a Zeiss SteREO Discovery.V8 microscope [28]. The area between the cemento-enamel junction (CEJ) and the alveolar bone crest (ABC) of molars 1, 2, and 3 of maxilla palatal, maxilla buccal, and mandible lingual sides of the jaw was measured in mm^2^ using Zeiss AxioVision software version 4.8.2 by a blinded viewer. The area between CEJ and ABC of the mandible buccal side cannot be measured because of occluding bone structure. Bone resorption was determined by comparing the area between the CEJ and ABC in infected to control mice. Intrabony defect was determined by as present or absent on the palatal and buccal sides of all three molars, and the percent was determined by dividing the number of intrabony defects by the total number of sites. Measurements represent the average bone resorption determined by three independent viewers blinded to the groups [29].

### Periodontal Inflammation

The mandibles and maxillas from 3 mice were collected on sacrifice and fixed in 10% neutral buffered formalin and decalcified with Immunocal (Decal Chemical Corporation, Tallman, NY) for 7 days at room temperature. Analysis proceeded as previously described [26]. Briefly, the decalcified tissues were embedded in paraffin and 5 µm sections were stained with hematoxylin and eosin (H & E). H & E stained slides were scanned at 20× using an APerio Scan Scope and examined using Aperio ImageScope v11.0.2.725.

### Antibody Analysis

Serum was collected on sacrifice and stored at −20°C until analysis could be performed. *P. gingivalis-*specific IgG and IgM antibody titer was determined by ELISA [30] using whole cell *P*. *gingivalis* as antigen. Serum was diluted 1∶100 while secondary goat anti-mouse conjugated to alkaline phosphatase (Bethyl Laboratories, Inc. Montgomery, TX) was used at a 1∶5000 dilution. Absorbance of each well was read at OD_405_ using a Bio-Rad Microplate Reader, and a standard curve was used to determine the titer.

### Localization of *P. gingivalis* by Fluorescence *in situ* hybridization (FISH)

FISH was used to detect bacteria that were metabolically active within tissues using probes specific to bacterial ribosomal 16S RNA [31]. FISH was performed on formalin-fixed paraffin embedded jaw and aorta tissue sections using a protocol modified from Fuchs [32] and Mantz [33]. Tissue sections were deparaffinized, blocked with Denhardt's reagent (Fisher Scientific, Pittsburg, PA), washed, and probed with 5 µg/ml of *P. gingivalis* 16S rRNA-specific oligonucleotide POGI [34] 5′-CAATACTCGTATCGCCCGTTATTC-3′ labeled with Alexafluor-568 (Invitrogen, Carlsbad, CA) in hybridization solution (900 mM NaCl, 20 mM Tris-HCl pH 7.5, 0.01% SDS, 20% formamide) for 3 h at 46°C. Following hybridizing of the probe, slides were incubated in wash buffer (20 mM Tris-HCl, pH7.5, 5 mM EDTA, 0.01% SDS, 0.225M NaCl), at 48°C for 25 min. Tissues were counter-stained with DRAQ5 (ThermoScientific, Asheville, NC) and mounted in Mowiol 4-88 (Sigma-Aldrich Corp, St. Louis, MO). Data were acquired at 63× on a Leica DMIRB microscope equipped with a Photometrics cascade-cooled EMCCD camera, controlled by the open-source software package µManager (http://www.micro-manager.org/). Images were processed using ImageJ (NCBI). Blocking buffer, hybridization buffer and wash buffer all contained protectRNA (Sigma-Aldrich, St. Louis, MO) to protect bacterial RNA from degradation.

### Atherosclerotic Plaque Analysis

Hearts (n = 6) and aortas (n = 6) were fixed in 10% neutral buffered formalin and embedded in paraffin. The heart at the level of the aortic valve and the adjacent ascending aorta as well as three equal length sections of the aortic arch, thoracic and abdominal aorta were harvested and 5 µm cross sections cut (two up to nine sections per aortic area, eight to thirty-six sections per mouse). Each aortic area was sectioned at 60–80 µm intervals where 5 µm sections were cut in order to reach a level where plaque was either detected or not detected (up to nine sections) and all were H&E stained for histological analysis. Minimal if any plaque was detectable in the thoracic and abdominal aortic sections, and therefore the aortic valve, ascending aorta and aortic arch areas with the greatest amounts of detectable plaque were utilized for the analyses of plaque size. Plaque area throughout the aorta from the level of the aortic valve to the descending, abdominal aorta as well as intimal/medial thickness ratios was assessed. The intimal-to-medial thickness ratio was used to normalize intimal thickness to changes in medial thickness seen at differing aortic levels. Plaque was measured using an Olympus DP71 microscope and ImagePro MC 6.0 software standardized to the microscopic objective. Plaque area, intimal thickness, medial thickness and calculated intimal/medial thickness ratios were evaluated using ImagePro software by a blinded reviewer. Intimal medial thickness ratios are used to reduce variations produced by differing vessel sizes.

### Aortic Inflammatory Cell Infiltration

Immunohistochemical staining was performed on aortic cross sections from mice infected for 12 weeks (n = 6) and 24 weeks (n = 6) and the corresponding control sham-infected mice (n = 6, both 12 and 24 weeks). F4/80-specific primary antibody (ab100790, Abcam, Cambridge, MA) was used to detect macrophages, and CD3-specific antibody (ab5690, Abcam, Cambridge, MA) was used to detect T cells, and all components of the developer kit are from the Abcam EXPOSE Rabbit specific HRP/DAB detection IHC kit (ab80437). Briefly, sections at the level of the aortic valve were deparaffinized, blocked with hydrogen peroxide, boiled for 20 min in citrate buffer, blocked with Abcam protein block, washed and covered with primary antibody diluted to working concentration (F4/80–1/100, CD3–1/100) in diluent (S3022, Dako, Carpinteria, CA), and incubated overnight at 4°C. Sections were then washed and incubated with secondary rabbit-specific HRP conjugate for 1 h. Sections were rinsed and developed with DAB, and nuclei stained with hematoxylin. Positive cells were counted in three high power fields (100X) for each section from each mouse by a reviewer blinded to the groups using ImagePro software.

### Lipid Profile, Oxidized LDL, Serum Amyloid A (SAA), and Endogenous NO Measurements

Serum was collected on sacrifice and the serum lipid profile, oxidized LDL (oxLDL), amyloid A, and NO measurements performed. Thirty microliters of serum from 24-week infected (n = 6) and control (n = 6) mice was sent for analysis of cholesterol and triglycerides by Skylight Biotech Inc (Akita, Japan). Serum oxLDL titers of infected (n = 6) and control (n = 6) mice were determined using the mouse serum oxidized low-density lipoprotein ELISA kit (TSZ ELISA, Waltham, MA). Levels of serum amyloid A (SAA) in 24 week-infected mice and controls were measured by colorimetric assay using an ELISA kit according to the manufacturer's instructions (Kamiya Biomedical, Seattle, WA). Titers were determined using a standard curve. Serum NO concentrations were measured using a NO fluorometric assay kit according to the manufacturer's instructions (Bio Vision Inc., Milpitas, CA).

### RT^2^ Profiler PCR Array

Expression of 84 genes known to be involved in pathogenesis of atherosclerosis was examined in aortas of infected (n = 3) and control (n = 3) mice by qRT-PCR with the RT^2^ Profiler Mouse Atherosclerosis PCR Array (SABiosciences, Valencia, CA). Tissues were homogenized by QIAGEN TissueRuptor (QIAGEN, Valencia, CA). RNA was extracted using an RNeasy kit (QIAGEN), followed by reverse transcription with the RT^2^ First Strand Kit (QIAGEN). RNA samples were prepared for array with the SYBR Green Master mix (QIAGEN). Cycling was performed following manufacturer protocol, and data were analyzed using the PCR Array Data Analysis V4 excel worksheet, downloaded from the SABiosciences website.

### RayBio Mouse Inflammatory Cytokine Array

Serum was collected from 12- and 24-week infected and control mice on sacrifice, pooled, and used to detect 40 cytokines on the Ray Biotech Mouse Inflammatory Cytokine Array (RayBiotech, Inc, Norcross, GA), according to the manufacturer's protocol. Briefly, sera was diluted 5-fold, and incubated overnight on glass chip arrays. The chip was washed, incubated 2 h with biotinylated secondary antibody, washed and incubated 2 h with streptavidin-conjugated fluorophore. Array slides were read with a GenePix 4400 scanner, using GenePix Pro 7.2.29.002 software. Results were analyzed using the RayBio Analysis Tool excel sheet.

### Statistical analysis

Statistical analyses of ELISA, serum lipid profile, SAA, NO, flow cytometry and horizontal alveolar bone resorption were performed using a two-tailed Student's t test with GraphPad Prism software v. 5, and *P*<0.05 were considered statistically significant. ELISA, flow cytometry, and horizontal alveolar bone resorption graphs show mean with standard deviation. Aortic histology measurements and immunohistochemistry cell counts were analyzed by ANOVA with the Statview program and *post hoc* PLSD analysis, and graphs are represented as mean with standard error.

## Results

### Chronic infection induced classical periodontal disease symptoms

*P. gingivalis* was detected in the oral cavity of 11 out of 24 mice by the third infection and in 12 out of 12 mice by the seventh infection (Table 1). Increasing numbers of *P. gingivalis*-infected mice throughout the study indicated that constant re-infection with *P. gingivalis* is necessary to establish a chronic oral infection in mice.

**Table 1:** Oral Microbial Samples.

		Positive Oral Microbial Samples						
		Total Mice/Plaque Sample#						
Bacterial Infection	24				12			
	1	2	3	4	5	6	7	8
P. gingivalis	0	0	11	0	0	11	12	12
Control	0	0	0	ND	ND	ND	ND	ND

By the third infection 45% of mice tested positive for *P. gingivalis* genomic DNA, and by the 7^th^ infection 100% of mice tested positive for *P. gingivalis* genomic DNA. ND – samples were not collected.

Alveolar bone resorption is the hallmark characteristic of PD. Twelve-week-infected mice had statistically significant alveolar bone resorption relative to uninfected mice in the maxilla palatal, maxilla buccal, and mandible lingual sides of the oral cavity (all *P*<0.001) (Figure 1B). Twenty-four week-infected mice also had statistically significant alveolar bone resorption relative to uninfected mice in all three measureable sides of the jaw (all *P*<0.001) (Figure 1B). Intrabony defects (vertical resorption away from the tooth root) are predictive of a poor prognosis in periodontitis patients. Intrabony defects were observed in 26% of sites in 12-week infected mice versus 9% of sites in controls, and in 21% of sites in 24 week-infected mice versus 5% in controls. The significant bone resorption in the infected mice and particularly the development of intrabony defects at more sites in infected mice indicates that the chronic oral infection with *P. gingivalis* effectively induces PD in mice.

Histological sections of mandibles and maxillae revealed minimal inflammation and little epithelial hyperplasia under conditions of infection at either 12 or 24 weeks (Figure 1D i, iii, respectively). In mice, *P. gingivalis* appears to poorly induce the gingival inflammatory response, which is a hallmark of PD and which in humans is responsible for initiating alveolar bone resorption, the definitive marker of PD.

FISH revealed metabolically active *P. gingivalis* within mandible and maxilla gingival tissues of both 12- and 24-week infected mice (Figure 1Ei and ii, respectively). Bacteria were visible, specifically in tissues surrounding each of the three molars. Frequently the bacteria were associated with locally proliferating inflammatory cell clusters in the connective tissue (Figure 1Ei), but were also seen within the gingival epithelia (Figure 1Eii). These results indicate that even in the presence of metabolically active *P. gingivalis* little oral inflammation is present in this murine model of PD.

**Figure 2:**
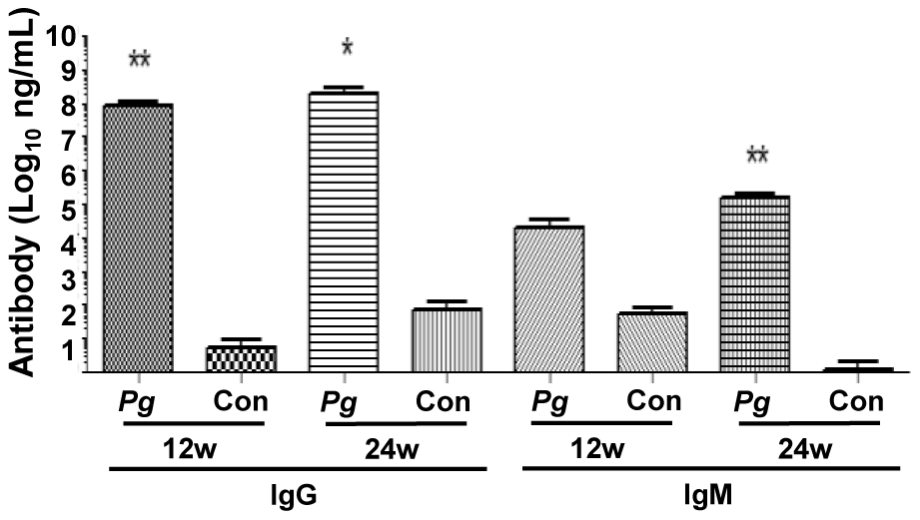
*P. gingivalis*-specific humoral immune response. Serum IgG and IgM titers were determined by ELISA. Both 12- and 24 week-infected mice (n = 6) had statistically significantly higher levels of IgG relative to controls (n = 6). Only 24 week-infected mice had significantly higher levels of IgM relative to controls. (*P<0.05, **P<0.01).

Systemic antibodies to *P. gingivalis* are clinically used as an indication of PD. Twelve-week-infected mice presented with significantly elevated levels of *P. gingivalis*-specific IgG when compared to uninfected mice (*P*<0.01) (Figure 2). Twenty-four-week-infected mice presented with significantly higher levels of *P. gingivalis*-specific IgG (*P*<0.05) and IgM (*P*<0.05) when compared to uninfected controls (Figure 2).

### Systemic spread of *P. gingivalis* and active invasion of the aorta

Oral bacteria are able to enter the blood stream from the mouth through microscopic tears in gingival tissues during common activities such as brushing, flossing, and dental procedures, resulting in low level, transient bacteremia. Thus, to evaluate whether local administration of *P. gingivalis* can result in systemic distribution of the organism, the presence of bacterial genomic DNA in multiple organs was evaluated. In the 12-week-infected group, *P. gingivalis* genomic DNA was detected in hearts and in aortas (Table 2). In the 24-week infection group, *P. gingivalis* genomic DNA was detected in hearts, aortas, livers, spleens, and kidneys (Table 2). Our results indicate that *P. gingivalis* is able to spread from the oral cavity to systemic organs.

**Table 2:** Systemic spread of *P. gingivalis* in internal organs.

Weeks	Bacterial Infection	Positive Systemic Tissue Samples					
		Number of Organs Sampled by PCR					
		Ht	Ao	Li	Sp	Ki	Lu
12	*P. gingivalis*	3	5	0	0	0	0
	Control	0	0	0	0	0	0
24	*P. gingivalis*	11	1	3	1	3	0
	Control	0	0	0	0	0	0

Systemic spread of *P. gingivalis* detected by presence of *P. gingivalis* genomic DNA by PCR in systemic organs. Ht-heart, Ao-aorta, Li-liver, Sp-spleen, Ki-kidney, Lu-lung. N = 12 Ht, Li, Sp, Ki, Lu; N = 6 Ao

**Figure 3:**
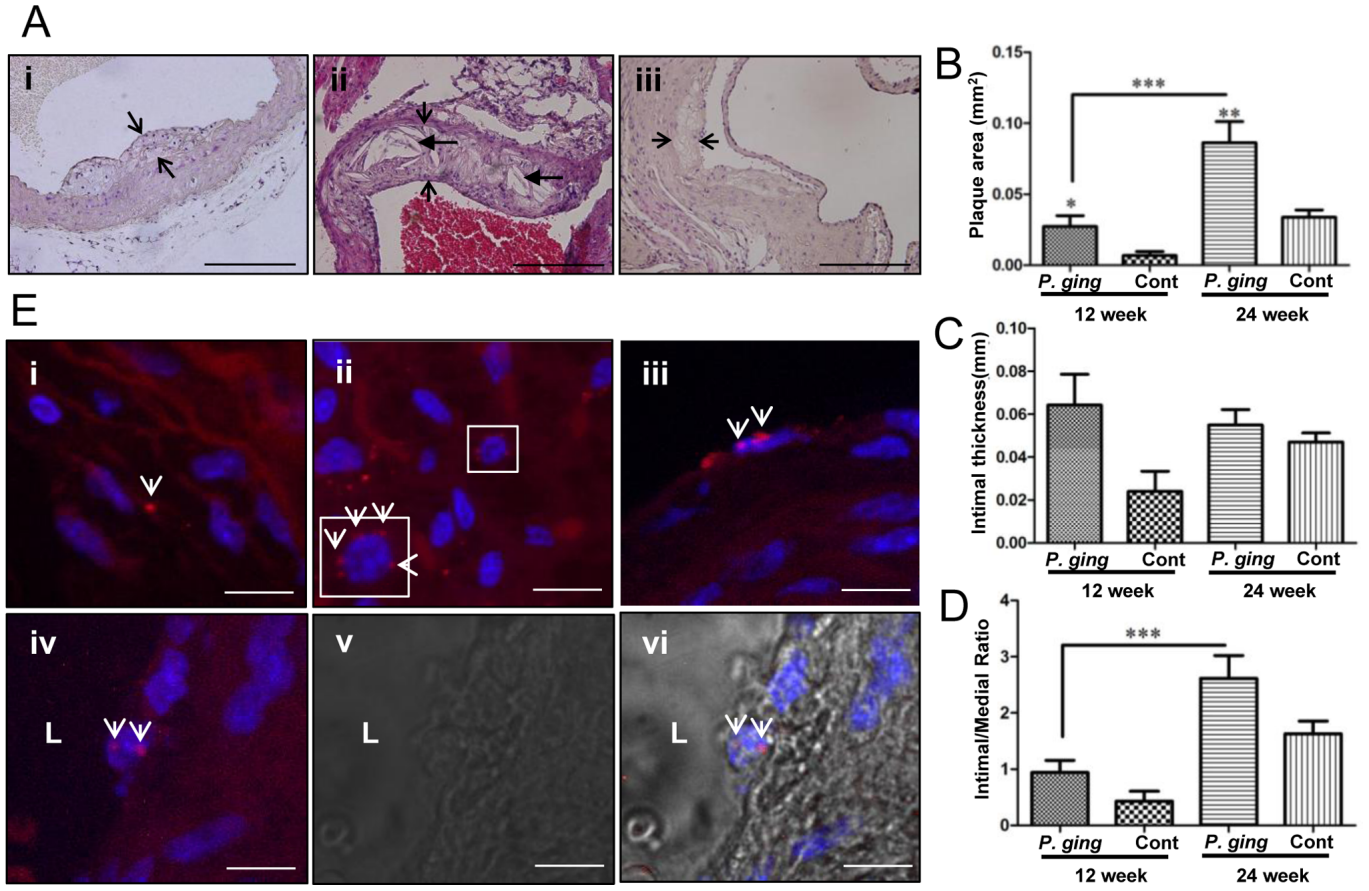
*P. gingivalis* infection-induced plaque growth in the aorta of mice at 12 and 24 weeks. FISH demonstrated viable bacteria in the aortic vessel concomitant with plaque growth. Hematoxylin and eosin stained aortic cross sections (A) i–12 weeks after *P. gingivalis* infection with demonstrated plaque in the mouse aorta; ii–24 week *P. gingivalis*-infected mouse ascending aorta with plaque containing cholesterol crystals; iii–12 week control aorta at the level of the aortic valve showing a small plaque. Arrowheads delineate intimal plaque margins, elongated arrows indicate cholesterol crystals. Scale bar represents 100 µm. Bar graphs of morphometric analysis of mean aortic plaque area (B), intimal thickness (C), and intimal/medial thickness ratios (D). (N = 12 for all graphs). Plaque area of 12 and 24 week-infected mice was significantly greater than controls, (*P*<0.05). (E), FISH illustrating *P. gingivalis* (red) in 12 week-infected mouse (i) aortic vessel, (ii) adventitia (perinuclear localization of *P. gingivalis* can be seen the inset), and (iii) a cluster of *P. gingivalis* in an endothelial cell. (iv) *P. gingivalis* in the endothelial cell of a 24-week infected mouse, (v) brightfield view of iv, (vi) overlay of iv and v. L – aortic lumen. Mouse cell nuclei are pseudo-colored blue. White arrows indicate labeled bacterial cells. Scale bar is 10 micrometers.

To assess the effects of chronic *P. gingivalis* periodontal infection on the development and progression of atherosclerotic plaque, aortic cross sections were examined histologically. Although both 12-week-infected mice and 24-week-infected mice had significantly greater mean aortic plaque area when compared to uninfected controls (*P*<0.05, and *P*<0.01, respectively) (Figure 3B), the intimal thickness and intimal/medial thickness ratio were not significantly elevated at either time point (Figure 3C, D). The largest plaques were consistently detected in the ascending aorta at or near the aortic valve. Cholesterol crystals were detected in 24-week-infected mice (Figure 3Aii), indicating accumulation of large amounts of oxidized lipoprotein particles. The plaque area and intimal/medial thickness ratio of 24 week-infected mice were statistically significantly greater than in the 12-week-infected mice, indicating that plaque progression is exacerbated in this chronic bacterial infection model.

Viable *P. gingivalis* were detected by FISH in the aortic wall, on the luminal side of the aortic wall, and within the adventitia of 12- (Figure 3Ei-iii) and 24-week-infected mice (Figure 3Eiv-vi). Together, these data suggest that the bacteria are actively invading the aortic wall and exacerbating development of the aortic plaque.

### Inflammatory cell infiltration of the aorta

**Figure 4:**
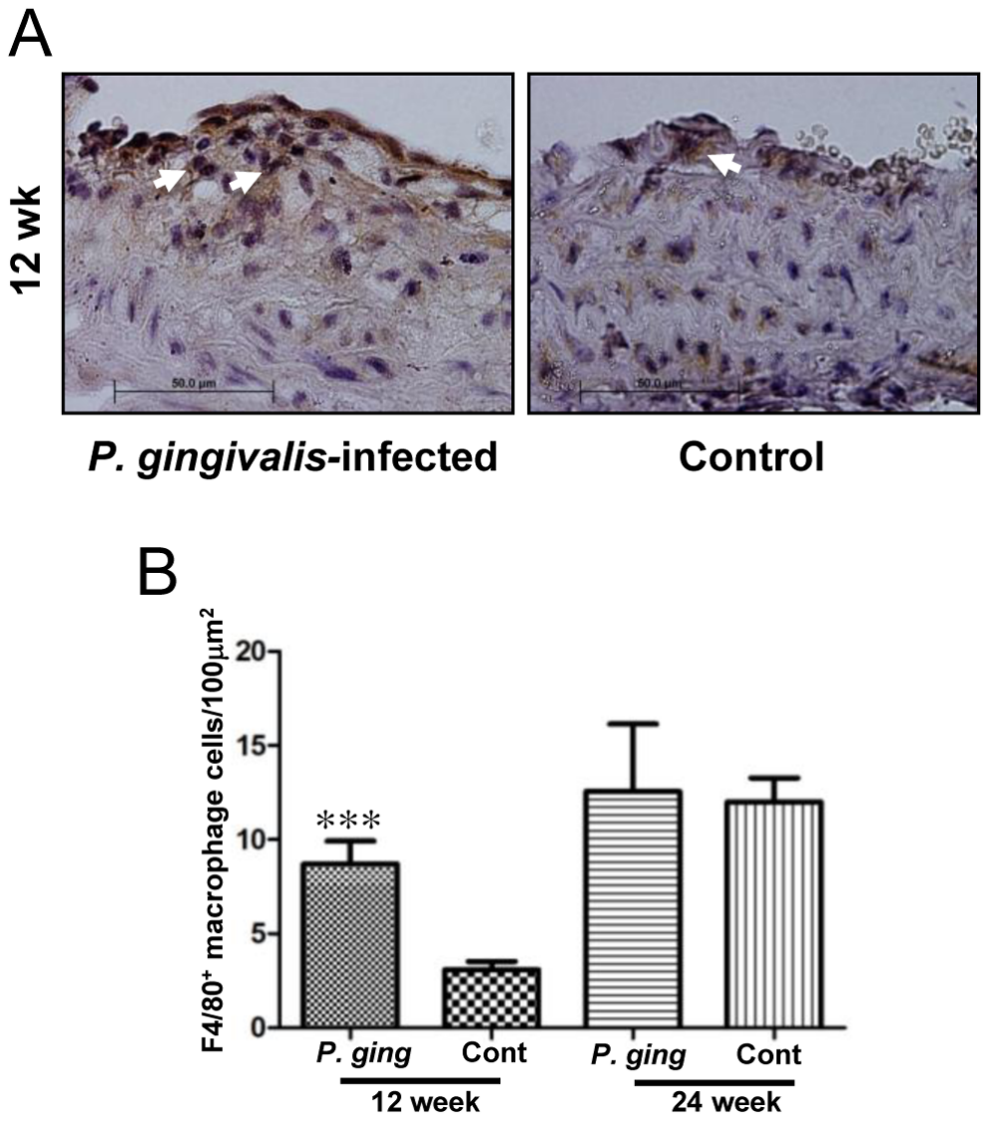
Macrophage invasion into the aortic arch intimal layer at 12 and 24 weeks of infection. Macrophage cell counts are significantly increased at 12 weeks follow up. (A), Immunohistochemical staining of aortic arterial cross sections demonstrate F4/80^+^ macrophage cells in 12-week-infected mouse and 12-week control mouse. White arrow heads indicate positively-stained cells. (B), Macrophage cell counts for each mouse represent counts in three high-power fields (100X) in the 12 and 24 week groups (***P<0.001). N = 6 for all groups.

To assess the local immune response to invading pathogens in the aorta, histological sections were stained to detect the major inflammatory cells present in atherosclerotic lesions, macrophages and T cells. The presence of macrophages and select classes of T cells in early lesions are reported to drive lesion development. We found that in the intimal layer of aortas from 12-week-infected mice, there were significantly elevated numbers of positively staining macrophages on immunohistochemical analysis when compared to 12-week sham-infected controls (*P*<0.001) (Figure 4A, 4B). However, there was no significant difference in the number of T cells present (not shown). In the twenty-four-week-infected mice, there was no difference in the number of macrophages (Figure 4B) nor T cells (not shown) present in the intima compared to twenty-four-week sham-infected controls.

### Systemic atherosclerosis risk factors and aortic gene expression changes

**Figure 5:**
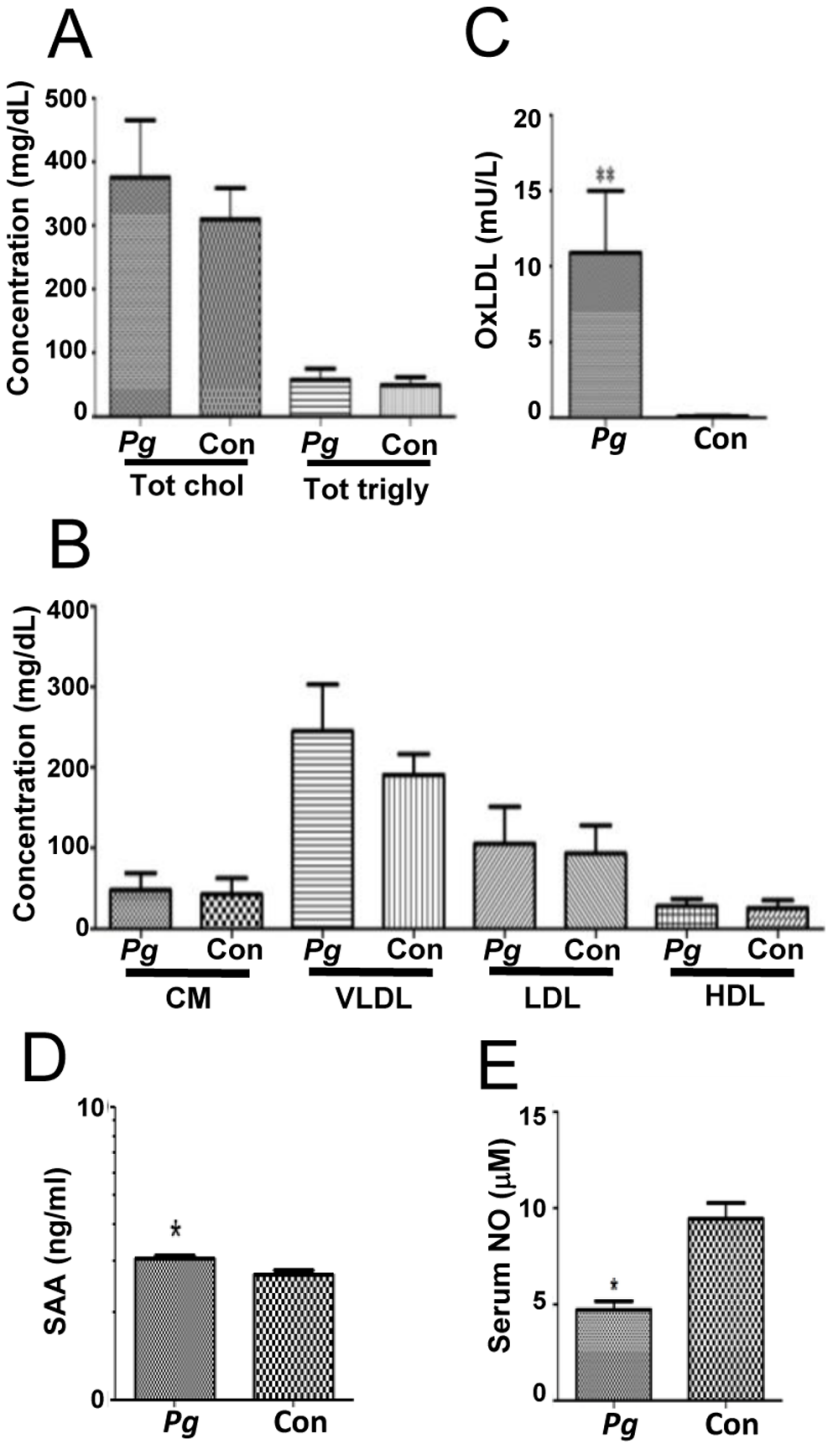
Systemic risk factors for atherosclerosis are elevated in *P. gingivalis* infection. (A, B), Graphical representation of Table 3 (n = 6). CM – chylomicron, VLDL – very low-density lipoprotein, LDL – low-density lipoprotein, HDL – high-density lipoprotein, Pg – *P. gingivalis*, Con – control. C, Level of oxidized LDL in serum of 24 week-infected mice is significantly higher than in controls (**P<0.01; n = 6). (D) Infected mice had statistically significantly elevated levels of SAA relative to controls. *P<0.05. E, Infection with *P. gingivalis* significantly reduced serum nitric oxide relative to uninfected control mice (*P<0.05; n = 6).

To evaluate the effect of systemic distribution of *P. gingivalis* on aortic health, systemic atherosclerotic risk factors were also evaluated. Overall, infected mice exhibited slightly higher levels of total cholesterol, which did not reach significance, along with triglyceride levels comparable to control mice (Figure 5A, Table 3). Similarly, very low-density lipoprotein (VLDL) particles were elevated in all infected mice, but again not to a level of significance. Levels of chylomicrons (CM), low-density lipoprotein (LDL), and high-density lipoprotein (HDL) particles were also comparable to control mice (Figure 5B, Table 3). On the other hand, 24-week-infected mice had statistically significantly elevated levels of oxidized LDL and serum amyloid A relative to controls (Figure 5C, D). Interestingly, mice infected with *P. gingivalis* also presented with significantly reduced (*P*<0.05) levels of circulatory NO (n = 6; 4.73±0.17 µM) compared to age-matched controls (n = 6; 9.45±0.24 µM) (Figure 5E). Together with elevated amyloid A, these data indicate an acute inflammatory response along with endothelial dysfunction during infection, and oxidized LDL levels correlate with greater plaque area.

**Table 3:** Serum lipid profile components of *P. gingivalis* infected mice.

Total	*P. gingivalis*-infected	Control	P-value
Cholesterol	375.09±90.62	309.97±48.45	>0.05
CM	47.72±20.98	42.46±20.16	>0.05
VLDL	244.93±57.84	190.71±25.87	>0.05
LDL	105.10±45.98	93.28±34.8	>0.05
HDL	27.83±9.10	25.39±9.8	>0.05
Triglycerides	57.09±18.07	49.08±12.69	>0.05

Values are in mg/dL ± SD. N = 6 in each group. Abbreviations are as follows: CM – chylomicron, VLDL – very low-density lipoprotein, LDL – low-density lipoprotein, HDL – high-density lipoprotein.

To evaluate infection-induced changes in the expression of genes involved in atherosclerosis development, a PCR array using aortas from 24-week-infected mice was performed. Genes noticeably affected by infection are listed in Table 4. A substantial decrease in the expression of blood clotting/coagulation cascade molecules was observed, along with an increase in expression of neuropeptide Y (*Npy*). A moderate change in expression of the Th2 response genes was also observed. Additionally, a moderate change in the expression of the inflammatory response genes was detected. The anti-apoptotic regulator gene *Birc3* was greatly increased while pro-apoptotic gene *Fas* was moderately decreased. Extracellular matrix molecules and cell-cell adhesion molecules were moderately affected. Numerous lipid transport and metabolism genes were down-regulated.

**Table 4:** Atherosclerosis-related gene expression changes upon 24 weeks of infection.

Gene grouping	Gene	Fold change
Apoptosis	*Birc3*	15.7
	*Fas*	−1.6
Blood clotting/coagulation cascade	*Serpinb2*	−4.32
	*Npy*	2.99
	*Fga*	−15.1
	*Fgb*	−11.6
Immune response	*Ccl5*	1.58
	*Ccr1*	−2.18
	*Ccr2*	−1.70
	*Csf2*	1.78
	*IL1β*	−1.51
	*Il3*	1.86
	*IL4*	1.66
	*IL5*	1.73
Leukocyte/endothelial cell adhesion	*Itgax*	2.33
	*Lama1*	3.66
	*MMP1α*	1.86
	*Selp*	−1.58
	*Thbs4*	−2.68
Lipid transport/metabolism	*Abca1*	−11.6
	*Apoa1*	−7.85
	*Apob*	−5.86
	*Fabp3*	−2.31
	*Ldlr*	2.10
	*MSR1*	−1.68
	*Plin2*	−1.35
	*Pparα*	−2.15
	*Pparγ*	−1.38

Twenty-four week infected and control aortic tissue samples were processed and analyzed as described in methods.

To examine the influence of infection on the levels of serum cytokines, an inflammatory cytokine array was performed. In 12-week-infected mice, 21 cytokines were altered in infected mice relative to controls (Table 5). Infected mice demonstrated a modest increase in the concentration of T cell chemoattractants as well as other leukocyte chemoattractants. On the other hand there was a modest decrease in additional lymphocyte chemoattractants in infected mice compared to controls.

**Table 5:** Serum cytokines altered by oral infection with *P. gingivalis.*

	12 week		24 week	
Cytokine grouping	Cytokine	Fold change	Cytokine	Fold change
Cell activation and proliferation	CD 30L	138	IL1α	1.7
	IL1α	2.5	IL1β	−3.4
	IL1β	−1.6	IL2	−2
	IL3	2.2	IL4	56
	IL4	146	IL6	−2.2
	IL9	2.1	IL13	15
	IL10	1.6	IL17	1.3
	IL-12p40/p70	2288		
	IL13	−17		
	CXCL12	−2.6		
Leukocyte chemoattractants	CXCL13	−5.8	CXCL13	−2.8
	CCL11	−21	CCL11	−16
	CXCL1	2.1	CXCL1	−4.1
	CXCL5	−4.9	CXCL5	−15
	CCL2	2.3		
T cell chemoattractants	CCL24	−9	CCL24	−2.1
	CX3CL1	−3.5	CX3CL1	1.3
	CXCL11	1.6	XCL1	1.3
	CSF1	1.9	CCL5	−1.6
	XCL1	1.9		
ECM molecules	TIMP-1	−1.8		
Apoptosis			FasL	−2.7

Sera from infected mice (n = 6) and control mice (n = 6) at both 12 and 24 weeks was analyzed as described in materials and methods. ECM – extracellular matrix.

In 24-week infected mice, fewer cytokines were detected at altered levels relative to controls (Table 5). Infected mice exhibited a modest increase in the concentration of T cell chemoattractants. A substantial decrease in eosinophil recruitment and activation cytokine CCL11, and neutrophil chemoattractants CXCL11 and CXCL5 was also observed. B cell chemoattractant CXCL13 was still decreased, yet B cell activation/maturation/proliferation cytokines IL4 and IL13 were greatly increased in infected mice.

## Discussion

Our assessment of periodontal disease in chronically orally infected mice indicates that the mice were colonized throughout the infection period. Although the mice do not exhibit the localized soft tissue inflammation that is seen in humans, they experience significant alveolar bone resorption, the major outcome of periodontal disease. There is also a strong systemic antibody response to *P. gingivalis*, which is further evidence of bacterial infection. A strong *P. gingivalis*-specific IgG response is also correlated with atherosclerosis in humans[35], which supports our data, that the humoral responses to *P. gingivalis* are not protective. In addition, viable *P. gingivalis* were detected by FISH within the gingival epithelia, indicating the invasive potential of this organism in the mouse.

Mounting evidence supports an active role for *P. gingivalis* in atherosclerotic plaque formation and progression. Based on comparison of atherosclerosis induction by wild-type *P. gingivalis* and a non-fimbriated mutant in ApoE^null^ mice, as well as earlier *in vitro* studies, Gibson, *et al*. [8], concluded that it is unlikely that *P. gingivalis* is only passively involved in atherosclerotic plaque formation. This conclusion is supported by clinical studies whereby *P. gingivalis* has been isolated from atherosclerotic plaque specimens [13]–[15]. Ours is the first study to demonstrate viable *P. gingivalis* by FISH within the vascular endothelial cells, aortic wall, and adventitia of orally infected mice. We were further able to demonstrate systemic dissemination of *P. gingivalis* by detecting genomic DNA within systemic organs. Together these data strongly support *P. gingivalis* dissemination and infection, particularly active infection of the aorta, as contributing factors in atherosclerotic plaque development.

In addition to or as a consequence of direct infection of the aortic tissues, oral infection by *P. gingivalis* alters many immunological processes that also exacerbate atherosclerotic plaque development. For instance, it is established that an imbalance in nitric oxide (NO) production could lead to oxidative stress and peripheral vascular diseases including atherosclerosis [23]–[25]. Our results are the first to demonstrate reduced circulatory levels of NO along with increased atherosclerotic plaque area in mice chronically orally challenged with *P. gingivalis*. Together these data suggest that either local infection with or dissemination of *P. gingivalis* causes an impairment of NO bioavailability, leading to endothelial-dependent vascular dysfunction.

High serum levels of low-density lipoproteins are also a risk for atherosclerotic plaque development, yet here we demonstrate that chronic *P. gingivalis* infection does not alter the serum lipid profile of ApoE^null^ mice. These results are consistent with results of others[8], strongly suggesting that *P. gingivalis*-induced atheroma formation is not due to altering the total amount of serum lipids, or the ratio of lipoprotein fractions. Further, the influence of *P. gingivalis* infection on atheroma formation is corroborated by the observation of a down-regulation of lipid transport and metabolism genes in the aortic tissues at 24 weeks of infection. Thus, it may be construed that *P. gingivalis* infection alters expression of the genes involved in atheroma formation intrinsically, but the role of any one particular lipid molecule in deciding the fate of atheroma formation is not clear.

Similarly, our PCR array and cytokine array revealed that expression of a select set of genes or proteins was altered in the aorta or peripheral blood, respectively, by oral infection with *P. gingivalis*. For instance, late stage aortic plaque rupture is influenced by the blood clotting/coagulation cascade, three components of which, *Fga*, *Fgb* and *Serpinb2*, were strongly down-regulated during infection with *P. gingivalis*, indicating a decreased tendency for blood clotting. This may help *P. gingivalis* to facilitate spread within aortic plaque, where fibrin deposition is common [36]. The decrease in expression of *Fas* and increase in *Birc3* indicate a potential suppression of apoptosis, which is supported by the decrease in FasL detected by the protein array at 24 weeks. This correlates with *in vitro* studies which have shown that *P. gingivalis* inhibits apoptosis of the cells which it has invaded [37].

Strong TH1 cell differentiation is evident in infected mice, which have a great increase in IL12p40/p70, and CD30L. Cytokines involved in T cell recruitment and activation was both moderately up- and down-regulated during chronic infection. As this is a chronic infection, it may be postulated that different T cell subsets are recruited throughout the infection period, and the cytokines activated reflect the functionality associated with specific T cell subsets. Our immunohistochemical staining did not reveal a difference in T cell counts between infected and control groups at either time point, but it is possible that the T cell subsets were altered between infection and control groups. By 24 weeks of infection, we observed fewer cytokines that were altered from the levels of uninfected mice, than were altered at 12 weeks of infection. This may be due to infection and inflammation reaching an equilibrium state by 24 weeks of infection. This is supported by the results of our macrophage staining in the mouse aortas, which demonstrated a significantly greater number of macrophages present in the early lesions of the 12-week infected mice compared to controls, but no difference in cell count between infected and control groups at 24 weeks of infection.

In summary, this study provides strong evidence for both direct and indirect involvement of *P. gingivalis* in atherosclerotic plaque formation using a physiologically relevant model of chronic oral infection which progresses to PD. It is well documented in animal models that infection with the periodontal pathogen *P. gingivalis* accelerates atherosclerotic plaque formation by modulation of the innate immune response [38]. However, until now, there is limited evidence that viable *P. gingivalis* is directly involved in atherosclerotic plaque formation. Our study is the first to present the comprehensive effects of *P. gingivalis*-induced chronic periodontal disease in atherosclerotic plaque formation in an ApoE^null^ mouse model, demonstrating a causal association.
